# Polyvinyl Alcohol/Chitosan-Ethyl Lauroyl Arginate Bilayer Films with Dual Surfaces: Improved Physicochemical Properties and Antimicrobial Properties

**DOI:** 10.3390/polym18121463

**Published:** 2026-06-11

**Authors:** Shaocheng Xu, Lei Zhong, Dongyang Jiang, Fuqi Wu, Wing Cheung Law, Chak Yin Tang, Fengwei Xie

**Affiliations:** 1 Guangxi Key Laboratory for Polysaccharide Materials and Modifications, School of Chemical Engineering, Guangxi Minzu University, Nanning 530006, China; xsc-intothesky@outlook.com (S.X.); leiwin@gmail.com (L.Z.); kechengziliao09@163.com (D.J.); 2 School of Science, Tianjin Chengjian University, Tianjin 300384, China; wwuu99@outlook.com; 3 Department of Industrial and Systems Engineering, The Hong Kong Polytechnic University, Hung Hom, Kowloon, Hong Kong, China; roy.law@polyu.edu.hk; 4 China-Hungary Belt and Road Joint Laboratory on Food Science, Chongqing Key Laboratory of Speciality Food Co-Built by Sichuan and Chongqing, College of Food Science, Southwest University, Chongqing 400715, China

**Keywords:** chitosan, polyvinyl alcohol, ethyl lauroyl arginate, antibacterial activity

## Abstract

In this study, polyvinyl alcohol (PVA) and chitosan (CS) were used as the base materials, and ethyl lauroyl arginate (LAE) as the antibacterial agent to prepare biodegradable bilayer composite films (P/C-L), whose properties compared with those of the monolayer films (P-C-L) of identical composition. Scanning electron microscopy (SEM) results revealed that the P/C-L films formed a compact microstructure with tight interlayer adhesion. Fourier transform infrared spectroscopy (FTIR) confirmed the presence of intermolecular hydrogen bonds within the P/C-L films without the formation of new chemical bonds, and X-ray diffraction (XRD) indicates that the crystallinity of the P/C-L films was dominated by that of PVA. P/C-L films exhibited a dual-surface structure with a hydrophobic CS layer and a hydrophilic PVA layer, broadening their potential application range. The P/C-L films demonstrated superior water resistance and light transmittance to the P-C-L films. When the LAE content increased from 0% to 10%, the P/C-L films displayed a more stable range of variation concerning visible light transmittance, water contact angle (CS layers), and moisture absorption than that of the P-C-L films, with the corresponding changing values being 86.86% to 62.09%, 96.79°to 72.46°, and 8.35% to 19.78%, respectively. Regarding antibacterial properties, the P/C-L films exhibited significantly enhanced activity across all LAE concentrations. Notably, P/C-L films at 2% LAE already outperformed P-C-L films at 4% LAE. At an LAE content of 10%, the inhibition zone diameters of the P/C-L films against *E. coli* and *S. aureus* reached 39.42 mm and 42.15 mm, which were 12.71 mm and 13.10 mm larger than those of the P-C-L films, corresponding to increases of 47.58% and 45.09%, respectively. In addition, both the P/C-L bilayer films and the P-C-L films could achieve complete biodegradation within 30 days under laboratory soil burial conditions. These findings suggest that P/C-L films show advantageous overall characteristics, highlighting their strong potential in the field of sustainable active food packaging.

## 1. Introduction

With the development of human society, petroleum-based plastics have permeated all aspects of people’s lives. Due to their excellent mechanical and barrier properties as well as low cost, they are commonly used in the field of food packaging. Specifically, plastics in the packaging industry alone account for 50% of the total global plastic production [1]. According to statistics, annual global plastic output has surged past 300 million tons in 2020 and is heading for 900 million tons by 2050 [2,3]. A critical issue is that plastics, which are difficult to degrade, are flooding into natural ecosystems, causing severe environmental pollution and endangering wildlife and humans [4]. On the other hand, the growth of foodborne microorganisms in food poses significant challenges to food safety and quality; it not only causes huge economic losses to the food industry but also leads to foodborne diseases that endanger human life [5]. Addressing these issues is urgent, and feasible strategies include replacing petroleum-based materials with biodegradable polymers and using antimicrobial additives to eliminate foodborne microorganisms [6,7].

Chitosan (CS) is a linear cationic polysaccharide obtained through the partial deacetylation of chitin [8], which also has the potential to improve water quality without adverse effects on corals [9]. Its exceptional properties—including excellent film-forming capacity, biocompatibility, antimicrobial activity, renewability and biodegradability—make it an optimal substitute for petroleum-based film materials in various eco-friendly applications [10,11]. Bin Darwish et al. fabricated modified ultrafiltration PVDF films using CS, which increased the removal rate of humic acid from 72.4% to 95.6% and demonstrated excellent anti-fouling properties [12]. However, pure CS films for food packaging are limited by their inherent brittleness, inadequate mechanical properties, and poor water barrier capability [13]. Studies indicate that blending CS with polyvinyl alcohol (PVA) could offset these shortcomings and improve the properties of the composite films [14]. PVA is a synthetic polymer and is mainly made by partial hydrolysis of polyvinyl acetate [15]. Its biodegradability, safety, strong film-forming and mechanical properties make it widely used in food packaging [16]. Pure PVA films are widely used yet have limitations: low ultraviolet (UV) shielding, high moisture absorption, weak bioactivity, and poor thermal stability [17]. The rich amino and hydroxyl groups on CS chains can form numerous hydrogen bonds with the hydroxyl groups on PVA chains, thus guaranteeing compatibility [18]. Studies have demonstrated that CS-PVA blend films exhibit a uniform structure as well as improved mechanical and barrier properties [19]. Recently, numerous novel composite materials have been designed due to the compatibility and synergistic effects between CS and PVA. Zhang and Guo [20] introduced CS and hydroxylated multi-walled carbon nanotubes to the surface of cotton fabrics, and then treated the cotton fabrics with hydrophilic PVA. The treated fabrics demonstrated superoleophobic characteristics and outstanding resistance to pollution [19].

Antimicrobial active packaging plays a pivotal role in ensuring food quality and safety [21]. The incorporation of antimicrobial agents during packaging production is intended to release these components from the films into food products, thereby preventing, reducing or eliminating contamination caused by pathogenic or food spoilage microorganisms [22]. Ethyl lauroyl arginate (LAE) is a cationic surfactant derived from lauric acid and arginine [23]. It exhibits potent and broad-spectrum antimicrobial activity, and can effectively inhibit Gram-negative bacteria, Gram-positive bacteria, fungi and yeasts even at low dosages [24]. LAE binds to the anionic phospholipid bilayer of cell membranes via electrostatic interactions, disrupts the membrane potential, and further inhibits cellular metabolism and inactivates cells [22]. Furthermore, LAE demonstrates chemical stability and antimicrobial activity within pH 3 to pH 7 [25]. In addition, it can be rapidly metabolized and hydrolyzed in the human body into natural components such as lauric acid, L-arginine and ethanol [26]. LAE has been approved as a safe and legal food additive by the U.S. Food and Drug Administration and the European Food Safety Authority [22]. It has been observed that composite films with LAE exhibit significant antimicrobial properties, which enhance food safety and extend product longevity [22].

Despite the fact that the monolayer structure (simple CS-PVA blend) incorporates certain advantages of each component, it also inherits some drawbacks, such as a deficiency in barrier properties and water resistance. To overcome these drawbacks of the monolayer structure, bilayer-structured biopolymer films are recognized as a solution. Layer-by-layer (LBL) solvent casting is used to prepare bilayer films in order to obtain beneficial properties from different film substrates, improving the properties of the films [27]. Zhuang et al. [28] noted in their article that bilayer films composed of CS and PVA typically demonstrate superior mechanical and barrier properties when compared to the same component monolayer films [27]. Via LBL, Wu et al. [29] prepared bilayer films consisting of PVA and gum, exemplifying excellent UV barrier properties, mechanical properties, and antimicrobial activity [28]. Recent studies have achieved the application of 3D printing to produce bilayer films endowed with multifunctional characteristics [30]. For example, Chen et al. [31] used 3D printing to prepare bilayer films composed of gelatin-PVA and corn starch-PVA, which possessed excellent thermal stability, water resistance, UV barrier ability, and antibacterial properties [30]. Similar results were reported by Luo et al. [32], who successfully prepared active bilayer CS-hydroxyethyl cellulose films with strong antibacterial properties by means of 3D printing [31].

This study hypothesizes that bilayer assembly can synergistically enhance the barrier properties and antibacterial properties of the membrane substrates. Based on this hypothesis, a novel bilayer composite film comprising a LAE-loaded CS layer and a neat PVA layer was developed. This paper not only clearly expounds the relationship between the structure and properties of such bilayer films, but also explores the interactions and mechanisms between the double layers. Furthermore, the mechanical properties, light transmittance, and water barrier capability of the bilayer films were evaluated at different LAE contents. To clarify the antimicrobial activity of the bilayer films, the in vitro antibacterial activity of the CS layer against foodborne pathogenic bacteria *E. coli* and *S. aureus* was evaluated. Finally, the biodegradability of the bilayer films was evaluated. The obtained data of these bilayer films were compared with those of monolayer films, aiming to provide theoretical guidance and technical support for the research and development of novel bilayer active composite films.

## 2. Materials and Methods

### 2.1. Materials

Chitosan (molecular weight: approximately 150 kDa; low viscosity: <100 mPa·s; degree of deacetylation: ≥90%) was purchased from Shanghai Puke Biotechnology Co., Ltd. (Shanghai, China). Polyvinyl alcohol (1750 ± 50, average molecular weight: 80 kDa) was obtained from Sinopharm Chemical Reagent Co., Ltd. (Shanghai, China). Glycerol (analytical grade) was purchased from Guangdong Guanghua Sci-Tech Co., Ltd. (Guangzhou, China). Ethyl lauroyl arginate (98%) was acquired from Bide Pharmaceutical Technology Co., Ltd. (Shanghai, China). Tryptic Soy Agar (TSA, 028074) and Tryptic Soy Broth (TSB, 024048) were purchased from Guangdong Huankai Microbial Sci-Tech Co., Ltd. (Guangzhou, China). *E. coli* (CSC(B)26003) and *S. aureus* (ATCC25922) were obtained from Shanghai Luwei Technology Co., Ltd. (Shanghai, China).

### 2.2. Preparation of P/C-L Bilayer Films and P-C-L Monolayer Films

The bilayer films were prepared by the LBL. First, PVA powder (4%, *w*/*w*) was dissolved in distilled water, and the mixture was heated and stirred in magnetic stirrers (Heat collecting constant temperature magnetic stirrer, DF-101S, Shanghai Lichen Bangxi Instrument Technology Co., Ltd., Shanghai, China) at 95 °C and 430 r/min for 1 h. Glycerol (5%, *w*/*w*) was then added, and stirring was continued for 15 min to obtain PVA casting solutions. CS casting solutions were prepared in a similar way; the water bath temperature and stirring speed were set to 80 °C and 860 r/min, following the addition of 2% (*w*/*w*) acetic acid and 5% (*w*/*w*) glycerol. Then, LAE at different concentrations (0%, 1%, 2%, 4%, 7%, 10%, *w*/*w*) was added to the CS solutions, followed by continuous stirring at 430 r/min for 30 min to prepare CS-LAE (C-L) solutions. Afterwards, both PVA and C-L solutions were treated with an ultrasonic Processor (Ultrasonic processor, Model KH-2890J, Kehai Inc., Weihai, China) at 20 kHz and 400 W in pulse mode (2 s on/1 s off) for a total processing time of 5 min at room temperature, so as to achieve a homogeneous distribution, and a vacuum pump (Circulating Water Vacuum Pump, SHB-111, Gongyi Yuhua Instrument Co., Ltd., Gongyi, China) was used to remove bubbles. Finally, 30 g of PVA solution was cast onto a polytetrafluoroethylene template (120 × 120 mm^2^) and dried at 40 °C to form the first layers. Subsequently, 30 g of C-L solution was cast onto the first layers to form the second layers, and the films were dried at 40 °C for 24 h to obtain bilayer films (P/C-L). The first layers are designated as the P-layers, and the second layers as the C-layers.

Similar to the preparation process of bilayer films, the monolayer films were also fabricated via solvent casting. CS and PVA casting solutions were mixed at a mass ratio of 2:3, and then LAE with different mass ratios was added. The mixture was further stirred at 55 °C for 30 min at 430 r/min to prepare a P-C-L mixed solution. Then, 60 g of each prepared P-C-L mixed solution was cast into a polytetrafluoroethylene mold and dried at 40 °C for 24 h to obtain monolayer films (P-C-L).

### 2.3. Characterization of the Composite Films

#### 2.3.1. Scanning Electron Microscopy (SEM)

The cross-sectional microstructure of the samples was observed using a field emission scanning electron microscope (FE-SEM, Supra 55, Carl Zeiss NTS GmbH, Oberkochen, Germany). Composite film samples with intact surfaces were selected and quenched in liquid nitrogen to obtain fracture cross-sections. The resulting fracture cross-sections were oriented upward and mounted on aluminum stubs. After gold sputtering under vacuum conditions, the observations were performed at an accelerating voltage of 2 kV with a magnification of 2000×.

#### 2.3.2. Fourier Transform Infrared Spectroscopy (FT-IR)

Infrared spectra in the range of 300–4000 cm^−1^ with a resolution of 8 cm^−1^ were obtained using a Fourier transform infrared spectrometer (Nicolette Magna 550II, GMI, Ramsey, MN, USA). First, the sample was ground into powder using a file, followed by drying in an oven for 6 h to eliminate moisture from the powder. Then, the powder was mixed with KBr at a ratio of 1:30, and after tableting, infrared spectral analysis was performed.

#### 2.3.3. X-Ray Diffraction (XRD)

X-ray diffraction (XRD) patterns were obtained using an X-ray diffractometer (MiniFlex 600, Rigaku, Tokyo, Japan) with a CuKα radiation source, operating at 40 kV and 15 mA. Samples (2.2 cm × 2.2 cm) were fixed on the glass slides, and the scanning angle was set to 3°~40° with a step size of 0.02°. The diffraction patterns were analyzed using the MDI Jade 6.5 software, and the crystallinity of each film was calculated using Equation (1):

CI = *S*_1_/(*S*_1_ + *S*_2_) × 100% (1)

where *S*_1_ refers to the area of the crystalline region, and *S*_2_ denotes the area of the amorphous region [33].

#### 2.3.4. Thickness and Mechanical Properties

The thickness was determined as the average of measurements taken at five random positions on each film using a digital micrometer (Jingyou Mould Co., Ltd., Dongguan, China). The tensile strength (MPa) and elongation at break (%) of the composite films were evaluated using the electronic universal testing machine (JDL, load cell 5000 N, Tianfa Instruments Co., Ltd., Yangzhou, China). This test was carried out with reference to the ASTM D882 standard method. Before testing, the preconditioned film samples (26 mm × 102 mm) were fixed between two grips, and their initial distance apart was set to 50 mm, and operated at a rate of 10 mm/min. Each result was the average of three independent measurements for films of each formulation.

#### 2.3.5. Light Transmittance and Opacity

An ultraviolet-visible spectrophotometer (UV-2600, Shimadzu, Kyoto, Japan) was used to measure the optical properties of composite films in a wavelength range from 200 to 800 nm. The films to be tested were cut into rectangular samples (12 mm × 40 mm), their UV barrier properties were assessed in the 200–400 nm range, and their visible light transmittance and opacity were assessed in the 400–800 nm range [34]. The opacity (Opa) of each film was calculated using Equation (2):

Opa = (−logT600)/H(2)

where T600 represents the transmittance at 600 nm, and H represents the average thickness of the film.

#### 2.3.6. Water Contact Angle (WCA)

The WCA of the composite films was measured by using a contact angle meter (OCA25, DataPhysics Instruments GmbH, Filderstadt, Germany). The film sample (20 mm × 50 mm) was fixed on a glass slide. 1 μL of distilled water was dropped onto the sample’s surface using a micrometer syringe. Subsequently, the contact angle between the water droplet and the sample was recorded by a high-definition CMOS camera. Each result was the average value of the film at five randomly selected points.

#### 2.3.7. Moisture Absorption (MA)

The sample to be tested (30 × 30 mm) was placed in a drying oven (DHG-9076A, Shanghai Jinghong Experimental Equipment Co., Ltd., Shanghai, China) at 40 °C until its weight achieved a constant value. At this point, the sample’s weight was noted as *M*_0_. Afterward, the sample was placed in a desiccator that contained a saturated NaCl solution and maintained a relative humidity of 75%. After 24 h, its weight was again noted as M_1_. The MA of each film was calculated using Equation (3):

MA = (*M*_1_ − *M*_0_)/*M*_0_ × 100% (3)

#### 2.3.8. Water Vapor Permeability (WVP)

The WVP of the composite films was measured following ASTM E96-00 [35] standard with some modifications [36]. The thickness of the tested film sample was measured at five different random positions before testing. The beaker, which had a volume of 50 mL and contained 30 mL of distilled water, was tightly sealed with the film sample and Vaseline. The sealed beaker was placed in a closed desiccator containing 1 kg of silica gel at 25 °C. The sealed beaker was weighed every 3 h until its weight remained constant. The WVP of each film was calculated using Equation (4):

*R =* Δ*m* × *H*/(*t* × Δ*P* × *S*) (4)

where *R* = the water vapor transmission (g/(m·s·Pa)), Δ*m* = change in weight of the entire testing beaker, *H* = the film’s thickness (m), *t* = the time interval (s), Δ*P* = a partial pressure gradient (2533 Pa), and *S* = the effective area for water vapor permeation (m^2^).

#### 2.3.9. In Vitro Antimicrobial Activity

The in vitro antibacterial activity of composite films with varying LAE contents was evaluated by the agar diffusion method according to Li et al. [37]. The composite films were cut into circular samples whose diameter was 8 mm, before the samples and tryptic soy agar (TSA) plates were sterilized for 30 min on a clean bench (ABC-4A1, Esco Micro Pte Ltd., Singapore). Next, 0.3 mL of *E. coli* was inoculated onto the TSA plates. Then, the samples were placed in the center of the TSA agar plates and incubated at 37 °C for 24 h. Finally, the diameter of the inhibition zones on each agar plate was measured three times, and the average value was taken as the criterion for evaluating the antibacterial effect. The antibacterial treatment for *S. aureus* was performed in a similar manner.

#### 2.3.10. Soil Burial Degradation Experiment

In accordance with the method described by Bhat et al. [38], the biodegradability of composite films was determined via the soil burial method. The Composite film samples (50 × 50 mm) were buried in flowerpots with natural soil at a depth of 10 cm. The soil moisture was maintained by spraying 10 mL of distilled water daily, and photographic records were taken every 3 days to observe the degree of degradation of the composite films.

#### 2.3.11. Statistical Analysis

The experimental data were expressed as mean ± standard deviation (*n* ≥ 3). Tukey’s test was used for significance analysis, and differences were considered statistically significant at *p* < 0.05. IBM SPSS Statistics 27 software was employed for analysis of variance. Origin 2024 software was utilized for graphing.

## 3. Results and Discussion

### 3.1. SEM Analysis

**Figure 1:**
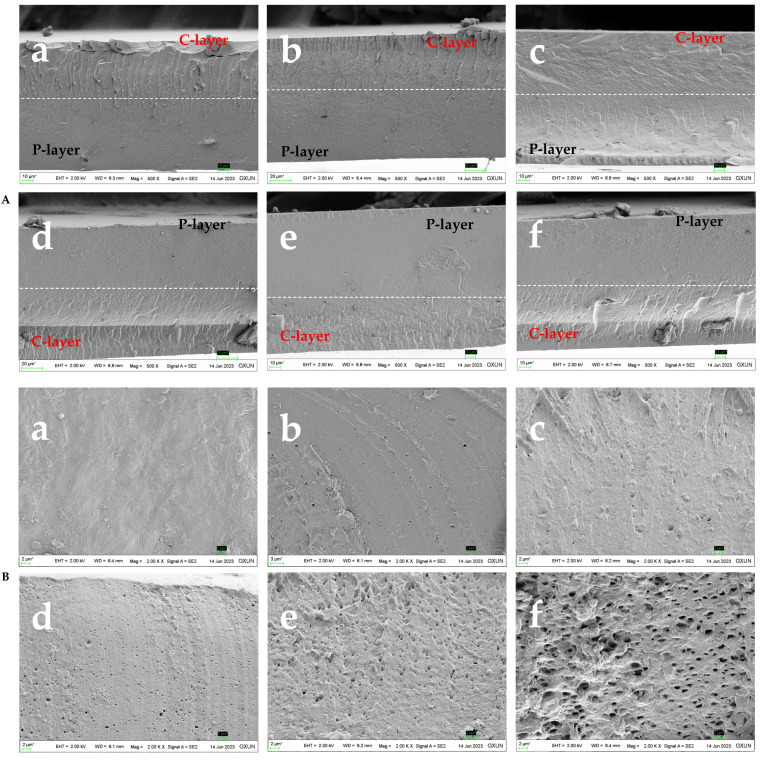
SEM images of P/C-L bilayer films (**A**) and P-C-L monolayer films (**B**) enriched with LAE (0–10%, *w*/*w*). Note: (**a**): No LAE added; (**b**–**f**): the addition levels of LAE are 1%, 2%, 4%, 7%, and 10% (*w*/*w*), respectively. The dotted line represents the interface between the C-layer and the P-layer.

The cross-sectional SEM images of P/C-L bilayer films with different LAE contents are shown in Figure 1. All images in Figure 1A exhibited a clear double-layer structure with a continuous and coherent interface. This phenomenon can be attributed to hydrogen bonding interactions at the interface and molecular chain entanglement between CS and PVA [29], which will be confirmed by the subsequent FTIR analysis. These results indicate the successful fabrication of the bilayer films. Notably, the C-layers appear rougher than the P-layers and exhibit obvious surface wrinkles. This is caused by the plane compression strain generated during the drying and shrinkage of the CS casting solution, which further leads to the deformation of the C-layers [39]. A similar phenomenon was also reported by Zhao et al. [40]. In addition, with an increase in LAE loading, the wrinkling degree of the C-layers gradually decreases, and the interface between CS and PVA becomes more uniform and coherent, suggesting that LAE facilitates improved interfacial compatibility between the two layers. In contrast, the monolayer films develop numerous pores when the LAE addition level is high. These differences likely arise due to two factors: first, as a surfactant, LAE reduces the surface tension of the casting solution, making it easier for air to enter and accumulate in large quantities in the film solution; second, the cross-linking effect between molecular chains, the viscosity of the CS-PVA blend is greatly increased, which inhibits the dissipation of bubbles and ultimately leads to the formation of a porous structure in the P-C-L monolayer film [41,42]. The relatively uniform and compact bilayer structure of the P/C-L films, in contrast to the porous morphology of the P-C-L films, is expected to contribute to superior water resistance, antibacterial activity, and light transmittance.

### 3.2. FTIR Analysis

**Figure 2:**
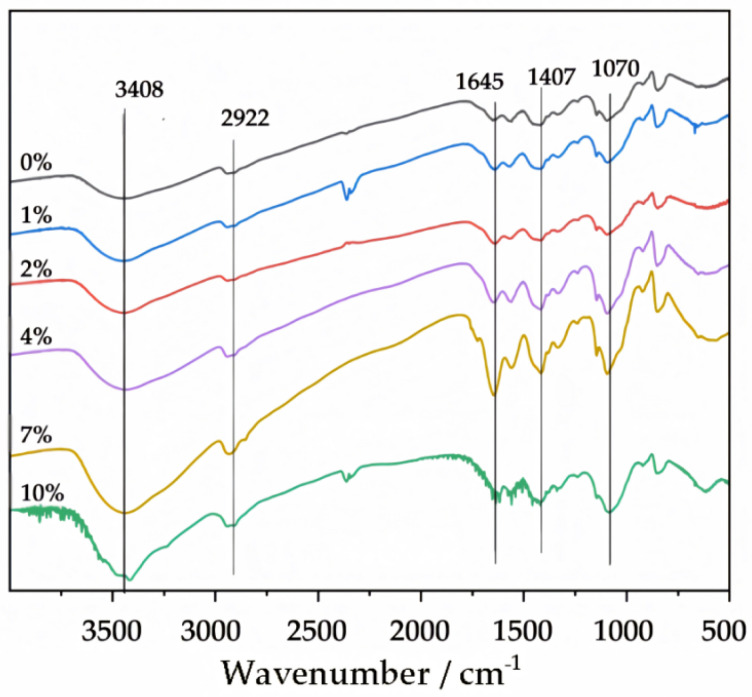
FTIR spectrum of P/C-L bilayer films enriched with LAE (0–10%, *w*/*w*).

The interactions between the components of the P/C-L bilayer films were analyzed via FTIR spectra, and the analytical results are shown in Figure 2. Peaks at approximately 1645 cm^−1^ and 1407 cm^−1^ are attributed to the amide I band (C=O stretching of acetylated amino groups) and amide III band (C-N stretching and N-H bending) of CS, respectively, which correspond to the characteristic absorption bands of CS [43,44]. The band at 1070 cm^−1^ is characteristic of PVA, which corresponds to the stretching vibrations of C-O bonds [45,46]. The broad absorption band at approximately 3408 cm^−1^ due to O-H stretching vibrations, and the absorption band at 2922 cm^−1^ is assigned to symmetric C-H stretching vibrations [47]. The O-H and N-H groups present in PVA and CS are capable of forming intermolecular hydrogen bonds [48,49]. The characteristic peaks of LAE highly overlap with those of the CS/PVA film matrix (3408 cm^−1^, 2922 cm^−1,^ and 1645 cm^−1^) [50], and the intensity of these characteristic peaks increased accordingly as the LAE content rose from 0% to 10%. This phenomenon indirectly confirms that LAE has been successfully incorporated into the film system. No new absorption peaks appeared in the infrared spectra following LAE incorporation, confirming that no new chemical bonds were formed between LAE and the film matrices, and that the interactions within the films were dominated by physical forces, including hydrogen bonds and van der Waals forces [51,52].

### 3.3. XRD Analysis

**Figure 3:**
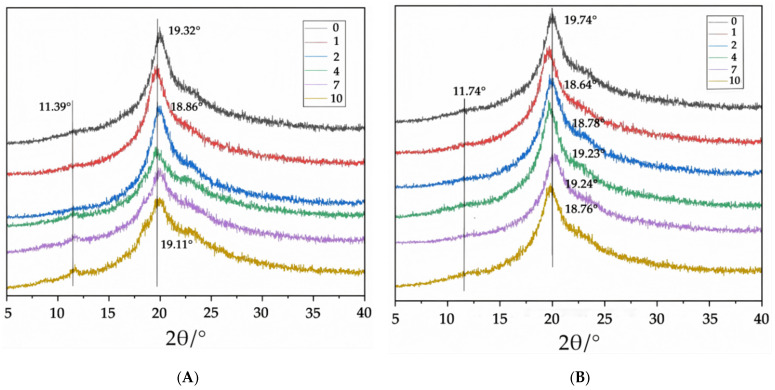
XRD patterns of P/C-L bilayer films (**A**) and P-C-L monolayer films (**B**) enriched with LAE (0–10%, *w*/*w*).

X-ray diffraction was employed to analyze the crystal types and crystallinity of P/C-L bilayer films with different concentrations of LAE, and the results are presented in Figure 3 and Table 1. A sharp diffraction peak of PVA was observed around 2*θ* = 19.2°, which exhibits the semicrystalline nature of the PVA films [53] and indicates the ordered arrangement of PVA polymer chains [54]; a weak diffraction peak of CS was observed around 2*θ* = 11.4°, which is the hydrated crystalline peak of CS [55] and reflects the random arrangement of the amorphous structure of CS molecular chains [56]. It is worth noting that the crystallinity of the P/C-L bilayer films gradually increases from 36.15% to 39.51% with increasing LAE addition amounts (1%, 2%, 4%, 7%, 10%, *w*/*w*), and no new diffraction peaks appear. This is because the incorporation of LAE changes the intermolecular forces of the bilayer films (the breaking and recombination of hydrogen bonds), promoting the formation of more ordered regions in the bilayer films and thereby enhancing their relative crystallinity [29]. A comparison of Figure 3A,B shows that both bilayer and monolayer films display intense PVA diffraction peaks and weak CS diffraction peaks, indicating that the crystalline structures of both films are dominated by PVA. Additionally, when combining the data in Table 1 and comparing the crystallinity of bilayer and monolayer films, it is found that the crystallinity values of the two films are similar with no significant difference (*p* > 0.05), indicating that the incorporation of LAE exerted little effect on the crystal structure of both film systems.

### 3.4. Thickness and Mechanical Properties

Thickness is a critical factor influencing key properties of the film, including mechanical properties, light transmittance, and water vapor transmission rate [57], with its values listed in Table 2. It can be seen that the incorporation of LAE did not influence the films’ thickness (*p* > 0.05). This might be because the low content of LAE is insufficient to significantly increase the average thickness of the films, as reported by Rubilar et al. [58]. Notably, the thickness of the bilayer films is consistently higher than that of the monolayer films, regardless of LAE addition. This may be because PVA interacts with CS via hydrogen bonds in the monolayer films, which shortens the distance between CS molecular chains and increases the film density [59].

**Figure 4:**
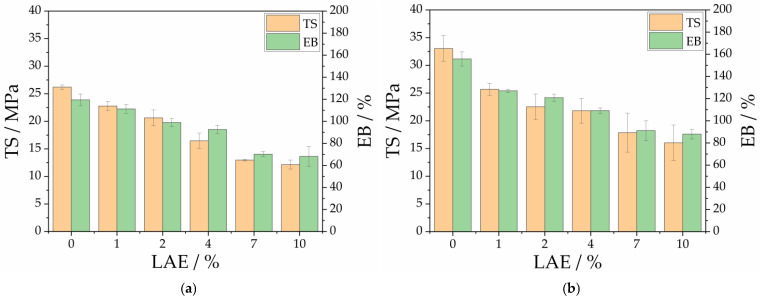
Mechanical properties of P/C-L bilayer films (**a**) and P-C-L monolayer films (**b**) enriched with LAE (0–10%, *w*/*w*).

Mechanical properties are crucial for evaluating the suitability of films as packaging materials. Most food packaging requires sufficient tensile strength (TS) and elongation at break (EB) to resist external stress and maintain an intact external structure [26]. Figure 4 and Table 2 illustrate the results of P/C-L bilayer films and P-C-L monolayer films on TS and EB. These experimental data indicate that the P/C-L films and P-C-L films have a similar variation in mechanical properties: both TS and EB decrease with the increase in LAE concentration. For the P/C-L films, the TS dropped from 26.20 MPa to 12.15 MPa with the incorporation of more LAE. This tendency might be due to the interactions between polar groups of CS and LAE, with the intermolecular network structure disrupted, and polymer cohesion in the films restricted [60]. Correspondingly, the EB decreased from 119.42% to 68.20%. This tendency could be explained by the fact that the agglomeration of LAE in the films enhanced molecular chain entanglement, which restricted the movement of CS macromolecular chains during deformation [61,62]. The TS and EB of the P/C-L films are lower than those of the P-C-L films. This phenomenon might be related to a mass of hydrogen bonds formed between CS and PVA molecular chains in the P-C-L films, and these hydrogen bonds enhance the cohesion of the polymer network in the film substrates [63].

Nevertheless, both film types retained adequate mechanical strength for food packaging applications [26]. Moreover, this reduction in mechanical performance should be considered in the context of the overall property profile: the bilayer architecture simultaneously imparts excellent water resistance and antibacterial activity, representing a balance between functional performance and mechanical integrity.

### 3.5. Light Transmittance and Opacity

**Figure 5:**
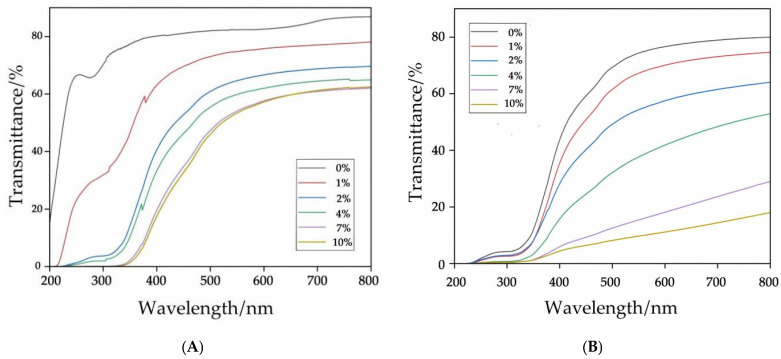
Light transmittance of P/C-L bilayer films (**A**) and P-C-L monolayer films (**B**) enriched with LAE (0–10%, *w*/*w*).

Materials with excellent UV shielding capability and high visible light transmittance are well-suited for food packaging. This is because they can effectively prevent the photo degradation of food products and maintain the sensory properties of food [64]. The spectral curves of the two types of composite films with different LAE contents in the full wavelength range of 200–800 nm are shown in Figure 5, and their light transmittance and opacity values at three characteristic wavelengths (200 nm, 400 nm, and 800 nm) are listed in Table 3.

The P/C-L bilayer control films showed a light transmittance of 15.01% at a wavelength of 200 nm, which indicates that the films do not possess UV resistance. When 1% LAE was incorporated into the bilayer films, the light transmittance in this wavelength region dropped significantly to 0.08%, exhibiting excellent UV-shielding capability. Furthermore, the light transmittance of the films decreased as the LAE content increased. It is speculated that the distribution and aggregation of LAE in the film network structure may increase light scattering, thereby hindering the light transmission process in the UV and visible light ranges [65]. Notably, the bilayer films containing 1% LAE still maintained a light transmittance of 78.12% at 800 nm, which showed high transparency, thus being suitable for food packaging.

Light transmittance and opacity of P/C-L bilayer films and P-C-L monolayer films with different LAE contents are compared in Table 3. It is found that the light transmittance of the bilayer films at the same wavelength is higher than that of the monolayer films after the addition of the same dose of LAE. Moreover, the bilayer films had good transmittance stability without a significant drop upon the addition of LAE. Taking the wavelength of 800 nm as an example, when the LAE addition increases from 0 to 10%, the transmittance of the bilayer films decreases from 86.86% to 62.09%, a drop of only 28.52%, while that of the monolayer films decreases from 79.95% to 18.23%, a drop of 77.20%. It is speculated that the PVA and CS molecular chains in monolayer films and LAE particles form a large number of aggregates through hydrogen bond cross-linking, and these aggregates alter the propagation direction of visible light, increase the difficulty of visible light transmitting through the monolayer films, and thus result in a higher decrease in light transmittance [66]. Regarding opacity, both film types showed increasing opacity with LAE addition, but the P-C-L films exhibited a substantially greater increase (1.71 to 14.35) compared with the P/C-L films (1.05 to 5.87), consistent with the transmittance results, confirming that LAE incorporation has a more pronounced effect on the optical clarity of the monolayer films.

Haghighi et al. [19] reported that the light transmittance of their prepared films decreased with the increase in LAE content, which was consistent with the effect of LAE on film transmittance in this study. After incorporating LAE, their films remained transparent in the visible light region (opacity < 5 at 600 nm). Nevertheless, the light transmittance at 400 nm was still higher than 50%, failing to achieve complete UV shielding effect. In addition, after Alkassfarity et al. [67] incorporated MCNC into their films, the UV shielding performance of the films was significantly improved, while their visible light transmittance decreased remarkably. Traditional films usually achieve efficient UV shielding at the expense of visible light transmittance. In contrast, the P/C-L bilayer films fabricated in the present study possess both superior properties. The P/C-L bilayer films can not only efficiently block ultraviolet light but also maintain excellent visible light transmittance, both of which are desirable properties for food packaging applications [68].

### 3.6. Water Contact Angle (WCA)

The higher the WCA on the surface of packaging films, the better their hydrophobicity [69]. The hydrophobic properties of packaging films not only contribute to maintaining their structural stability in humid environments but also effectively inhibit microbial growth on the films’ surfaces.

**Figure 6:**
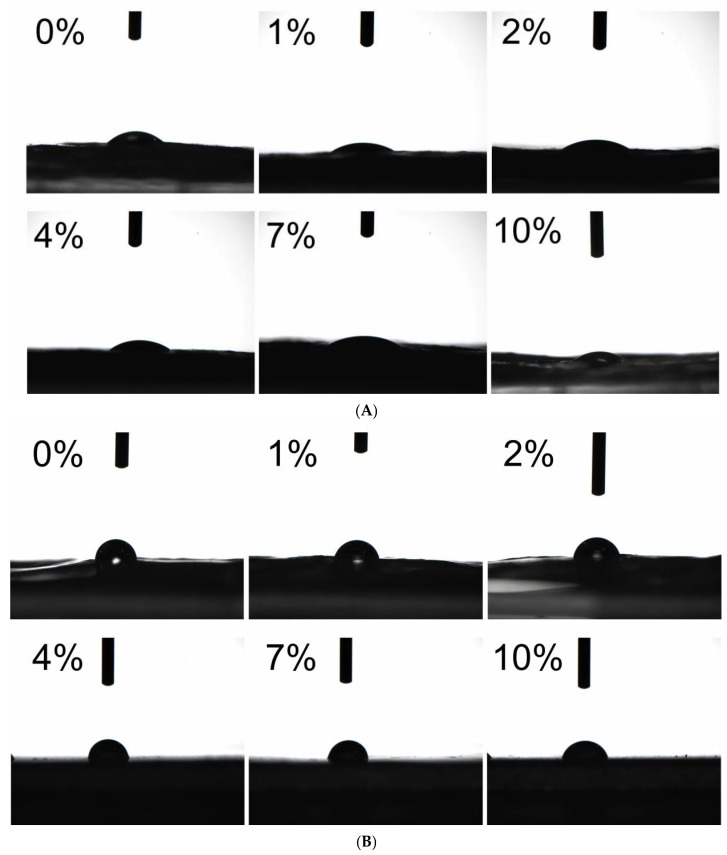
Water contact angles of the P-layers (**A**) and the C-layers (**B**) of P/C-L bilayer films enriched with LAE (0–10%, *w*/*w*).

To broaden their potential applications, the bilayer films were designed with two distinct surfaces: one hydrophilic and the other hydrophobic. In contrast, the monolayer films can only exhibit one of the two properties—either hydrophilic or hydrophobic. The WCA values of both bilayer and monolayer films with varying LAE contents are presented in Figure 6 and Table 4. Analysis of the results showed that adding LAE (1–10%, *w*/*w*) reduced the water contact angles of both films, but the decrease is more significant in monolayer films—from 91.10° to 22.64°—transforming them directly from hydrophobic to hydrophilic. In contrast, the bilayer films exhibit much more stable properties in WCA: their hydrophobic layer decreases from 96.79° to 72.46° and remains hydrophobic, while their hydrophilic layer dropped from 46.32° to 26.98°, with a much smaller decrease than that of the monolayer films. Li et al. [70] reported a similar phenomenon: the WCA of CS/SA bilayer films (98.92°) was obviously higher than that of CS (84.75°) and SA (40°) monolayer films, and the addition of ICDH reduced the hydrophobicity of the films; nevertheless, when the addition amount of ICDH reached 10%, the WCA of the CS layer in CS/SA bilayer films sharply decreased to 46.49°, which transformed the films from hydrophobic into hydrophilic ones. On the contrary, Du et al. [71] found that incorporating functional additives into CG/CS-GEL bilayer films could increase the WCA values, while such films failed to possess both hydrophilic and hydrophobic layers simultaneously.

The possible reason for the reduced WCA of both films is that the incorporated LAE increases polar groups such as free amino groups in the films, leading to more hydrophilic groups on the film surfaces, which results in a decrease in the films’ WCA [72]. The difference in the extent of WCA reduction may be attributed to two factors: on one hand, the presence of LAE in monolayer films creates pores, increasing the contact area between the film surfaces and water; on the other hand, the interfacial interaction between the C-layers and P-layer of bilayer films aggregates free hydrophilic groups, reducing the number of hydrophilic groups such as hydroxyl groups exposed on the film surfaces [73].

In short, the two sides of the C/P-L bilayer films possess hydrophilicity and hydrophobicity respectively, which is different from films that only have single hydrophilic or hydrophobic properties. Moreover, they exhibit better stability after incorporating functional fillers, thus showing prominent application advantages in the field of functional food packaging.

### 3.7. Moisture Absorption (MA)

**Figure 7:**
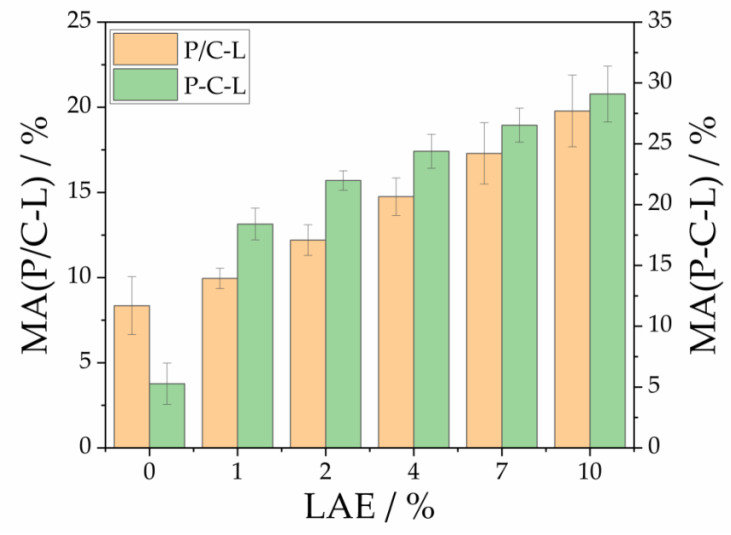
Moisture absorption rates of P/C-L bilayer films and P-C-L monolayer films enriched with LAE (0–10%, *w*/*w*).

MA is a key indicator of the water resistance in food packaging films. Films with high moisture absorption can swell, partially dissolve, and exhibit reduced barrier and mechanical properties during prolonged contact with water [74]. The MA of P/C-L bilayer films and P-C-L monolayer films are presented in Figure 7 and Table 5. The data makes clear that the addition of LAE leads to a gradual increase in the MA of both bilayer and monolayer films. Specifically, the MA value of bilayer films rose from 8.35% to 19.78%, while that of monolayer films increased from 5.27% to 29.09%. This phenomenon may be attributed to the high hydrophilicity of LAE, which exhibits a low oil-water partition coefficient (less than 0.1) and readily binds to water molecules [34]. Du et al. [71] also found a similar rule in their research. After incorporating flavonoid nanoemulsions into the prepared CG/CS-GEL bilayer films, the MA of the films increased from 29.30% to 38.60%, which was much higher than that of P/C-L bilayer films in this study, suggesting that the CS/PVA-based bilayer system exhibits superior resistance to moisture absorption.

In particular, the MA of the P/C-L films are generally lower than those of the P-C-L films under equivalent concentrations of LAE. In the absence of LAE, the MA of P/C-L films was 45.32% lower than that of the P-C-L films, and at 2% LAE content, the P/C-L exhibit a 44.99% lower MA than the P-C-L films. One of the possible factor for this phenomenon is the strong interactions between the P and C layers, which make hydrophilic groups aggregate at the interface and thus reduce the number of hydrophilic groups that can bind with water molecules [26]. Another possible factor for this phenomenon is that the incorporated LAE increases the size and number of pores in the monolayer films, and these pores provide spatial pathways for water penetration [75].

In summary, the P/C-L bilayer films loaded with LAE display a smaller growth range of moisture absorption. It is confirmed that the bilayer structure can effectively weaken the water absorption effect derived from functional additives, thus endowing the bilayer films with better water resistance. The low MA properties of the bilayer films are conducive to improving their durability and functional stability in humid food packaging environments.

### 3.8. Water Vapor Permeability (WVP)

WVP, a critical indicator for assessing moisture transfer across films, should be minimized to reduce moisture exchange between food products and the ambient atmosphere, thereby preventing food spoilage [26]. The WVP data of P/C-L bilayer films and P-C-L monolayer films are presented in Table 5. It was found that the WVP of the bilayer films ranged from 2.15 × 10^−10^ to 2.92 × 10^−10^ g·m^−1^·s^−1^·Pa^−1^ (*p* > 0.05), indicating that the incorporation of LAE barely affects their WVP. Gao et al. [76] noted in their study that HL-EOs (a mixed emulsion of hydroxypropyl-β-cyclodextrin, LAE, and essential oils) significantly reduced the WVP of active CS films, and they speculated that this was because small amounts of the emulsion not only increased the tortuosity of molecular pathways but also weakened the bonding between chitosan and water molecules, thereby reducing water permeability [67]. On the contrary, Ma et al. [77] found that the incorporation of LAE increased the WVP of CS-based films, attributing this to the disruption of intermolecular and intramolecular hydrogen bonds in CS by LAE molecules, which resulted in a looser film structure and thus increased water molecule channels [68]. These conflicting results suggest that WVP is influenced by various factors such as film fabrication processes, film substrates, and the type and concentration of antimicrobials.

The data in Table 5 explain that at elevated LAE content, the WVP of the P/C-L films generally showed a trend of decreasing, whereas that of the monolayer films increased progressively. When the LAE concentrations exceed 2%, the WVP value of the monolayer films surpasses that of the bilayer films. At 10% LAE concentration, the WVP of the bilayer films is 43.12% lower than that of the monolayer films. This behavior can probably be caused by two factors: on one hand, the thicker P/C-L bilayer films lengthen the diffusion path of water molecules [47]; on the other hand, LAE incorporation causes the reorganization of hydrogen bonds in the P-C-L monolayer film system, disrupting the long-range ordered structure between CS-PVA molecules and weakening their resistance to water molecule diffusion [19].

In summary, the bilayer films can not only maintain lower water vapor permeability, but also effectively offset the adverse effects of high-content LAE on the water vapor barrier property of films. This breaks through the limitation that high dosages of antibacterial agents tend to deteriorate the barrier performance of monolayer packaging films, and provides a novel strategy for the fabrication of high-barrier active food packaging films.

### 3.9. In Vitro Antimicrobial Activity

**Figure 8:**
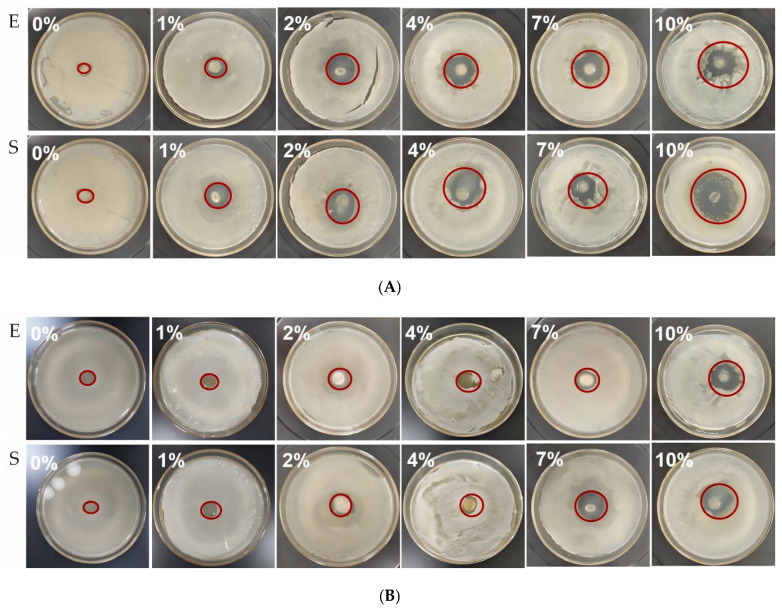
Inhibitory effects of the C-layers of the P/C-L films (**A**) and the P-C-L monolayer films (**B**) enriched with LAE (0–10%, *w*/*w*) on *E. coli* (E) and *S. aureus* (S). Note: The positions marked by red circles in the figure indicate the inhibition zones.

Foodborne microorganisms can cause food spoilage and foodborne diseases; thus, active films with antibacterial properties provide significant safeguards for foods. Table 6 presents data on the inhibition zone diameters of the C-layers of the P/C-L bilayer films and the P-C-L monolayer films. Their antibacterial efficacies are illustrated in Figure 8.

In this study, LAE was exclusively loaded onto the C-layer of the bilayer films. This strategy was designed considering that CS possesses inherent antibacterial activity. As both CS and LAE are cationic compounds, they are expected to exert a synergistic antibacterial effect [78]. Without the addition of LAE, the C-layers exerted a certain inhibitory activity against both bacterial strains. This is attributed to the interaction between the positively charged amino groups of CS and the negatively charged cell membranes, which leads to the leakage and expulsion of intracellular proteins, resulting in cell death [79]. When the LAE loading content in the C-layers increased from 0% to 10%, the inhibition zone diameters against *E. coli* increased from 9.32 mm to 39.42 mm, with an increment of 322.96%. Meanwhile, those against *S. aureus* rose from 10.63 mm to 42.15 mm, an increase of 296.52% (*p* < 0.05). This indicates that LAE greatly improved the antibacterial activity of bilayer films against the two strains, and its inhibitory effect increased with increasing LAE content. Similar phenomena were also reported by Dong et al. [52], whereas the maximum inhibition zone diameters of their films against *E. coli* (7.93 mm) and *S. aureus* (12.47 mm) were far smaller than the data in this study.

Furthermore, the data in Table 6 demonstrate that, after adding LAE, the antibacterial effect of the C-layers of the bilayer films against both *E. coli* and *S. aureus* was significantly superior to that of the monolayer films. At 10% LAE content, the diameter of the inhibition zone of both film types reaches their maximum values. Under this condition, the inhibition zone diameters of the bilayer films against *E. coli* and *S. aureus* are 47.58% and 45.09% larger, respectively, than those of the monolayer films. Notably, the C-layers of the P/C-L films at a LAE content of 2% already exhibited larger inhibition zones against both *E. coli* (25.84 mm) and *S. aureus* (26.54 mm) than the P-C-L films at 4% LAE (16.33 mm and 21.34 mm, respectively). Aseela Banu et al. [80] also confirmed in their research that bilayer films possessed better antibacterial activity than monolayer films. Nevertheless, the maximum inhibition zone diameters of their films against *E. coli* (13.00 mm) and *S. aureus* (14.00 mm) were far lower than those obtained in the present paper. LAE is released more rapidly in films with stronger water absorption and swelling capacity [22]. CS features a high swelling coefficient and can rapidly absorb water and swell upon exposure to moisture, while hydrogen bonds formed between CS and other polymer matrices can restrain this swelling behavior [81]. Numerous hydrogen bonds exist in the blended film system of CS and PVA [82]. Therefore, when the agar diffusion method is adopted to determine the inhibition zone diameter of films, the C-layer of bilayer films instantly absorbs water and swells, thereby accelerating the release of LAE and consequently presenting larger inhibition zone diameters.

The above results demonstrate that the bilayer structure enables the targeted enrichment of LAE. LAE is only loaded into the layer with inherent antibacterial activity, achieving the rapid release of LAE successfully. This strategy not only endows bilayer films with remarkably stronger antibacterial activity than monolayer films and those reported in existing studies, but also achieves superior antibacterial efficacy with a lower dosage of LAE, which provides a novel blueprint for the development of high-performance active food packaging materials.

### 3.10. Soil Burial Degradation

**Figure 9:**
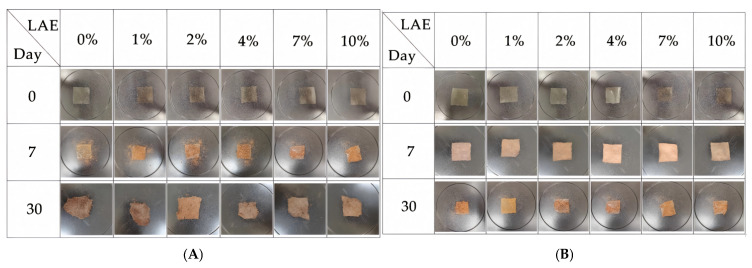
Soil burial degradation of the P/C-L bilayer films (**A**) and the P-C-L monolayer films (**B**) enriched with LAE (0–10%, *w*/*w*).

Compared with petroleum-based packaging films, bio-based packaging films offer a distinct environmental advantage due to their biodegradability. The degradation effects of the P/C-L bilayer films and the P-C-L monolayer films are shown in Figure 9A and Figure 9B, respectively. It can be seen that the degradation rate of the bilayer films was not significantly different from that of the monolayer films, and both were largely unaffected by LAE concentration. Within the first 7 days, the bilayer films exhibited increased surface roughness accompanied by visible cracks, which progressively intensified with time. After 30 days, some portions of the films were nearly fully disintegrated. Previous studies [83] have pointed out two stages of soil burial degradation: in the first stage, the film substrate absorbs water and swells, and microorganisms begin to adhere to the film surface; in the second stage, microorganisms multiply in large numbers, enzymatic hydrolysis breaks molecular bonds, and molecular chains are degraded into CO_2_ and H_2_O, leading to a sharp increase in degradation rate. These results demonstrate that both film types are capable of complete biodegradation within 30 days under laboratory soil burial conditions, confirming their potential as environmentally sustainable alternatives to conventional plastic packaging materials.

## 4. Conclusions

In this study, CS and PVA were selected as the main materials of films, adding LAE as the antibacterial agent. P/C-L bilayer films were prepared via layer-by-layer solvent-casting. The microstructure, intermolecular interactions, and comprehensive properties of the P/C-L bilayer films were investigated. SEM, FTIR, and XRD revealed that the film components interacted with each other through hydrogen bonds rather than forming new covalent bonds, and the phases were evenly distributed. The P-layers and C-layers in the bilayer films are well integrated, suggesting good compatibility. Compared with the P-C-L monolayer films, the P/C-L films had a more compact microstructure. The WCA values of both film types decreased with LAE addition (0–10% *w*/*w*), but the bilayer films show a smaller reduction. The bilayer P/C-L films preserved a hydrophobic C-layer (WCA ≥ 72.45°) and a hydrophilic P-layer (WCA ≤ 46.32°), a dual property that lays a structural foundation for expanding their application scenarios in functional food packaging. Under the same LAE content (0–10% *w*/*w*), the water barrier properties of the bilayer films were significantly better than those of the monolayer films, making them much better in preventing moisture transfer. Both the P/C-L bilayer films and the P-C-L monolayer films display good UV shielding capability, and bilayer films exhibit higher visible light transmittance and improved optical clarity. The antibacterial effect of the P/C-L bilayer films is greater. P/C-L films at 2% LAE already outperformed the P-C-L films at 4% LAE, demonstrating that the bilayer architecture achieves superior antibacterial efficacy at substantially lower additive loadings. At 10% LAE content, inhibition zones against *E. coli* and *S. aureus* were 47.58% and 45.09% larger, respectively, than those of the P-C-L monolayer films. Both film types started to degrade within 7 days and could achieve complete biodegradation within 30 days. Overall, the P/C-L bilayer films exhibited comprehensive improvements across structural, optical, barrier and antibacterial properties, highlighting their strong potential for application in active food packaging. However, this study has not investigated the release kinetics of LAE, the long-term antibacterial stability under real food storage conditions, the mechanical performance of the films under dynamic loading, and the durability of the films in practical application scenarios, which deserve further exploration.

## Figures and Tables

**Table 1:** Crystallinity of P/C-L bilayer films and P-C-L monolayer films enriched with LAE (0–10%, *w*/*w*).

LAE/%	P/C-L Bilayer Films/%	P-C-L Monolayer Films/%
0	36.15 ± 2.24 ^a^	36.66 ± 0.91 ^a^
1	36.89 ± 1.24 ^a^	37.05 ± 0.74 ^a^
2	38.39 ± 0.63 ^a^	37.50 ± 0.22 ^a^
4	39.00 ± 0.69 ^ab^	37.74 ± 1.49 ^a^
7	39.02 ± 0.65 ^ab^	37.91 ± 1.64 ^a^
10	39.51 ± 0.28 ^b^	38.06 ± 1.03 ^a^

Note: The mean values in the same column of the table with different superscript letters indicate significant differences in Tukey’s test at *p* < 0.05.

**Table 2:** Thickness and mechanical properties of P/C-L bilayer films and P-C-L monolayer films enriched with LAE (0–10%, *w*/*w*).

Film Sample	LAE/%	TS/MPa	EB/%	Thickness (mm)
P/C-L	0	26.20 ± 0.40 ^a^	119.42 ± 5.35 ^a^	0.170 ± 0.029 ^a^
	1	22.77 ± 0.83 ^b^	111.22 ± 3.95 ^a^	0.171 ± 0.033 ^a^
	2	20.63 ± 1.46 ^b^	98.89 ± 3.75 ^b^	0.171 ± 0.035 ^a^
	4	16.46 ± 1.40 ^bc^	92.57 ± 3.85 ^b^	0.172 ± 0.034 ^a^
	7	12.96 ± 0.14 ^cd^	70.20 ± 2.36 ^bc^	0.173 ± 0.035 ^a^
	10	12.15 ± 0.80 ^cd^	68.20 ± 8.96 ^bc^	0.174 ± 0.035 ^a^
P-C-L	0	33.03 ± 2.31 ^a^	155.81 ± 6.43 ^a^	0.160 ± 0.015 ^a^
	1	25.68 ± 1.08 ^b^	127.06 ± 1.47 ^b^	0.161 ± 0.014 ^a^
	2	22.54 ± 2.30 ^b^	120.84 ± 3.13 ^c^	0.161 ± 0.016 ^a^
	4	21.82 ± 2.25 ^b^	109.18 ± 2.58 ^d^	0.162 ± 0.010 ^a^
	7	17.86 ± 3.52 ^bc^	91.13 ± 8.91 ^e^	0.162 ± 0.014 ^a^
	10	16.03 ± 3.19 ^bc^	87.98 ± 4.46 ^e^	0.165 ± 0.018 ^a^

Note: The mean values in the same column of the table with different superscript letters indicate significant differences in Tukey’s test at *p* < 0.05.

**Table 3:** Light transmittance and opacity of P/C-L bilayer films and P-C-L monolayer films enriched with LAE (0–10%, *w*/*w*).

Film Sample	LAE/%	T200/%	T400/%	T800/%	Opacity
P/C-L	0	15.01	80.11	86.86	1.05 ± 0.06 ^a^
	1	0.08	64.18	78.12	1.35 ± 0.11 ^b^
	2	0.01	40.61	69.66	2.42 ± 0.30 ^c^
	4	0	33.25	64.92	4.24 ± 0.71 ^d^
	7	0	16.41	62.51	5.54 ± 0.35 ^e^
	10	0	15.77	62.09	5.87 ± 0.39 ^e^
P-C-L	0	0.04	46.64	79.95	1.71 ± 0.08 ^a^
	1	0	38.2	74.7	3.73 ± 0.36 ^b^
	2	0.02	30.2	64.2	4.86 ± 0.24 ^b^
	4	0	17.1	53.14	6.14 ± 0.89 ^bc^
	7	0	6.47	29.32	11.47 ± 2.23 ^bc^
	10	0.01	4.73	18.23	14.35 ± 0.54 ^bcd^

Note: The mean values in the same column of the table with different superscript letters indicate significant differences in Tukey’s test at *p* < 0.05.

**Table 4:** Water contact angle data of P/C-L bilayer films and P-C-L monolayer films enriched with LAE (0–10%, *w*/*w*).

LAE/%	P/C-L		P-C-L
	WCA (C-Layers)/°	WCA (P-Layers)/°	WCA/°
0	96.79 ± 3.32 ^a^	46.32 ± 3.52 ^a^	91.10 ± 3.51 ^a^
1	86.32 ± 3.80 ^b^	43.71 ± 2.64 ^ab^	48.53 ± 3.30 ^b^
2	83.38 ± 3.14 ^b^	41.45 ± 3.65 ^ab^	37.55 ± 2.18 ^bc^
4	82.83 ± 5.56 ^b^	38.38 ± 3.31 ^bc^	34.04 ± 2.34 ^bc^
7	77.31 ± 3.86 ^bc^	33.55 ± 2.86 ^c^	26.73 ± 2.20 ^bcd^
10	72.46 ± 2.66 ^bc^	26.98 ± 2.36 ^bd^	22.64 ± 1.90 ^bcd^

Note: The mean values in the same column of the table with different superscript letters indicate significant differences in Tukey’s test at *p* < 0.05.

**Table 5:** The MA and WVP data of P/C-L bilayer films and P-C-L monolayer films enriched with LAE (0–10%, *w*/*w*).

Film Sample	LAE/%	MA/%	WVP/g·m^−1^·s^−1^·Pa^−1^
P/C-L	0	8.35 ± 1.7 ^a^	2.87 × 10^−10^ ± 1.94 × 10^−11 a^
	1	9.95 ± 0.6 ^a^	2.92 × 10^−10^ ± 5.17 × 10^−11 a^
	2	12.20 ± 0.9 ^b^	2.39 × 10^−10^ ± 5.76 × 10^−12 a^
	4	14.75 ± 1.1 ^b^	2.27 × 10^−10^ ± 1.87 × 10^−12 a^
	7	17.29 ± 1.8 ^bc^	2.62 × 10^−10^ ± 9.00 × 10^−12 a^
	10	19.78 ± 2.1 ^cd^	2.15 × 10^−10^ ± 2.25 × 10^−11 b^
P-C-L	0	5.27 ± 1.7 ^a^	1.37 × 10^−10^ ± 1.22 × 10^−11 a^
	1	18.40 ± 1.3 ^b^	1.77 × 10^−10^ ± 2.98 × 10^−11 a^
	2	21.98 ± 0.8 ^bc^	2.39 × 10^−10^ ± 1.37 × 10^−11 b^
	4	24.38 ± 1.4 ^bc^	2.87 × 10^−10^ ± 5.12 × 10^−11 bc^
	7	26.52 ± 1.4 ^cd^	3.23 × 10^−10^ ± 2.60 × 10^−11 bc^
	10	29.09 ± 2.3 ^cd^	3.78 × 10^−10^ ± 5.62 × 10^−11 bc^

Note: The mean values in the same column of the table with different superscript letters indicate significant differences in Tukey’s test at *p* < 0.05.

**Table 6:** Diameters of antimicrobial zones for the P/C-L bilayer films and P-C-L monolayer films enriched with LAE (0–10%, *w*/*w*).

Film Sample	LAE/%	*E. coli*/mm	*S. aureus*/mm
C-layer of P/C-L	0	9.32 ± 0.25 ^a^	10.63 ± 0.25 ^a^
	1	15.43 ± 0.28 ^a^	22.42 ± 0.22 ^b^
	2	25.84 ± 0.19 ^c^	26.54 ± 0.18 ^c^
	4	28.33 ± 0.41 ^d^	32.13 ± 0.21 ^d^
	7	29.96 ± 0.32 ^d^	30.81 ± 0.29 ^e^
	10	39.42 ± 0.34 ^e^	42.15 ± 0.28 ^f^
P-C-L	0	11.32 ± 0.25 ^a^	11.87 ± 0.28 ^a^
	1	12.06 ± 0.38 ^b^	14.92 ± 0.25 ^b^
	2	14.04 ± 0.29 ^bc^	18.85 ± 0.38 ^bc^
	4	16.33 ± 0.31 ^bcd^	21.34 ± 0.27 ^bcd^
	7	19.52 ± 0.37 ^e^	25.38 ± 0.39 ^e^
	10	26.71 ± 0.34 ^f^	29.05 ± 0.24 ^f^

Note: The mean values in the same column of the table with different superscript letters indicate significant differences in Tukey’s test at *p* < 0.05.

## Data Availability

The original contributions presented in this study are included in the article. Further inquiries can be directed to the corresponding authors.
